# Supplementary material for: Legionella pneumophila exploits the endo-lysosomal network for phagosome biogenesis by co-opting SUMOylated Rab7
Source: bioRxiv. 2023 Oct 31:2023.10.31.564884. Preprint. [Version 1] doi: 10.1101/2023.10.31.564884 (PMC10634985; doi:10.1101/2023.10.31.564884)
Supplement: Supplement 1 [file NIHPP2023.10.31.564884v1-supplement-1.pdf]

bioRxiv preprint doi: https://doi.org/10.1101/2023.10.31.564884; this version posted October 31, 2023. The copyright holder for this preprint (which was not certified by peer review) is the author/funder, who has granted bioRxiv a license to display the preprint in perpetuity. It is made available under aCC-BY 4.0 International license.

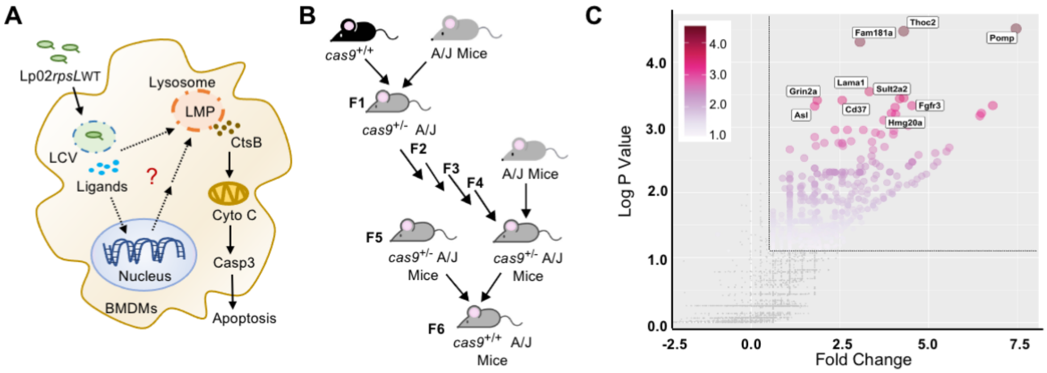

Figure S1 Identification of host factors that restrict Lp02*rpsL*$_{WT}$ replication in BMDMs via CRISPR/Cas9 knockout screenings

(A) Model of the lysosomal macrophage death triggered by infections with strain Lp02*rpsL*$_{WT}$. Ligands released by the bacterium trigger lysosomal membrane permeabilization and apoptosis in BMDMs, resulting in the termination of bacterial replication.

(B) Generation of *Cas9*$^{+/+}$ mice with A/J background. *Cas9*$^{+/+}$ mice with B6 background were breeding with A/J mice, their off-springs carrying heterozygous *Cas9*$^{+/-}$ gene were backcrossed with A/J mice for an additional three generations. *Cas9*$^{+/+}$ mice with A/J background were generated by mating between *Cas9*$^{+/-}$ A/J mice.

(C) Volcano plot of significant genes from samples containing large LCVs vs those containing small LCVs during CRISPR/Cas9 knockout screenings. Threshold: Fold change > 1.5, -log10 p value > 1, (*P* value <0.1). The 10 genes with the highest changes were highlighted

bioRxiv preprint doi: https://doi.org/10.1101/2023.10.31.564884; this version posted October 31, 2023. The copyright holder for this preprint (which was not certified by peer review) is the author/funder, who has granted bioRxiv a license to display the preprint in perpetuity. It is made available under aCC-BY 4.0 International license.

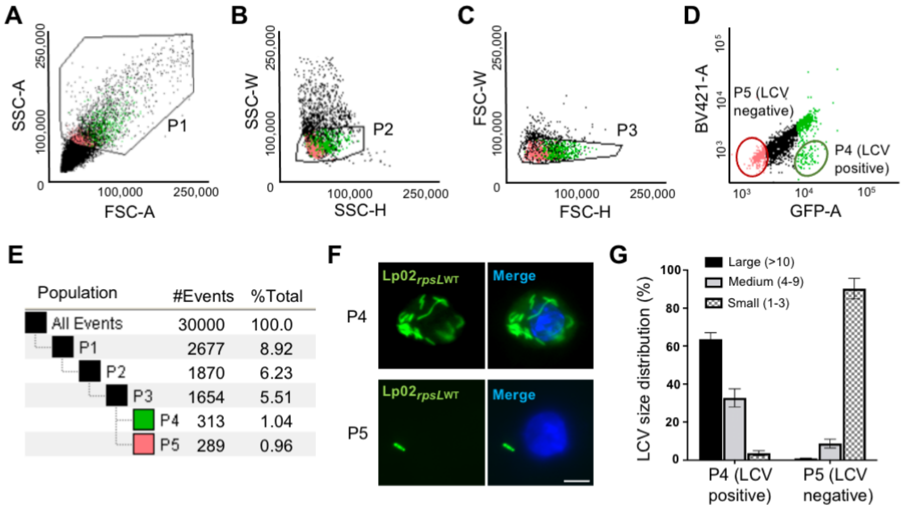

Figure S2 Sorting of BMDMs containing large LCVs by flow cytometry

(A) Forward scatter vs size scatter. Samples with estimated mouse macrophage size, complexity and infected by strain Lp02*rpsL*$_{WT}$(pGFP) were collected. Cell debris and dead cells were excluded from the sorting flow.

(B) Size scatter heights vs weights. Single macrophages were collected. Aggregated cells were excluded from sorting flow.

(C) Forward scatter heights vs weights. Cells stacked together were excluded from the sorting flow.

(D) GFP signal vs background signal. BV421 was set as the background control to separate the signal away from macrophage autofluorescence. P4 represents macrophages containing large LCVs while P5 represents macrophages containing only one bacterium.

(E) Percentage of cells in each portion. P4 or P5 accounts for 1% of total cell samples being examined.

(F) Representative images of macrophages containing large LCVs in P4 or small LCVs in P5 sorted by flow cytometry. DNA was stained with Hoechst. Bar, 5 μm.

(G) LCV size distribution of macrophages in P4 and P5 portions. At least 300 LCVs were scored for each sample.

bioRxiv preprint doi: https://doi.org/10.1101/2023.10.31.564884; this version posted October 31, 2023. The copyright holder for this preprint (which was not certified by peer review) is the author/funder, who has granted bioRxiv a license to display the preprint in perpetuity. It is made available under aCC-BY 4.0 International license.

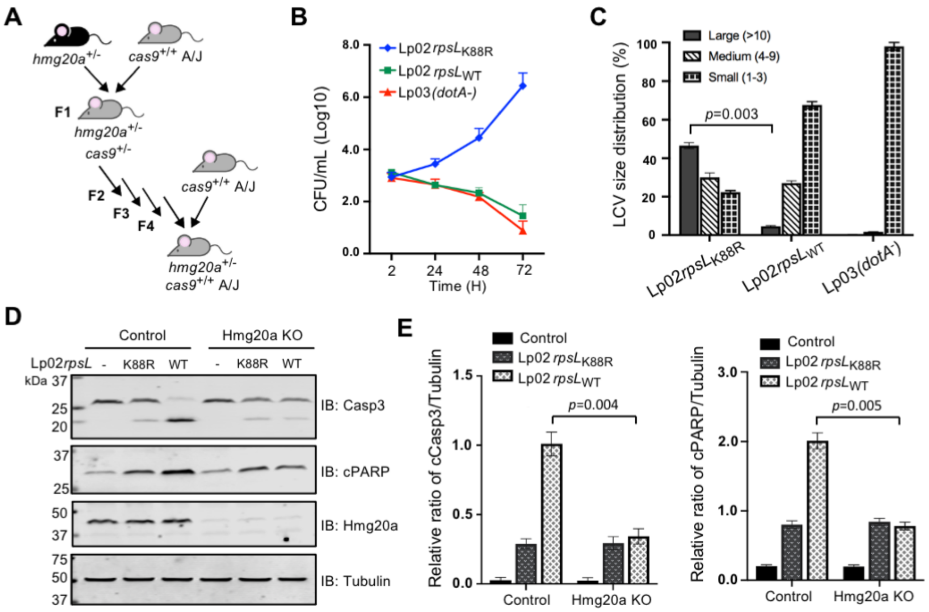

Figure S3 Generation of *Hmg20a*$^{+/-}$ *Cas9*$^{+/+}$ mice in A/J background

(A) *Hmg20a*$^{+/-}$ mice in B6 background were bred with *Cas9*$^{+/+}$ A/J mice, and their off-springs with the *Hmg20a*$^{+/-}$ *Cas9*$^{+/-}$ genotype were bred with *Cas9*$^{+/+}$ A/J mice for an additional four generations. *Hmg20a*$^{+/-}$ *Cas9*$^{+/+}$ mice with A/J background were generated by mating *Hmg20a*$^{+/-}$ *Cas9*$^{+/-}$ A/J mice with *Cas9*$^{+/+}$ A/J mice.

(B) Intracellular replication of relevant *L. penumophila* strains in BMDMs from *Hmg20a*$^{+/-}$ *Cas9*$^{+/+}$ A/J mice. BMDMs were infected with bacteria at an MOI of 0.05. Samples were plated at indicated time points to obtain total colony-forming units. Data represents mean ± s.e. from three experiments each done in triplicate.

(C) The distribution LCVs of different sizes formed by the indicated *L. penumophila* strains in BMDMs from *Hmg20a*$^{+/-}$ *Cas9*$^{+/+}$ A/J mice. BMDMs were infected by indicated strains at an MOI of 1 and infection were proceeded for 14 h before fixation and analysis.

(D) Protein levels of apoptosis-associated proteins in *Hmg20a* knockout BMDMs infected by relevant *L. penumophila* strains. Cells were lysed 8 hpi and processed for immunoblotting using antibodies specific for caspase3, cleaved RARP, Hmg20a or Tubulin.

(E) The relative ratio of the band intensity of cleaved caspase3, cleaved RARP to Tubulin. Date shown as mean ± s.e. of relative ratio calculated from three independent experiments.

bioRxiv preprint doi: https://doi.org/10.1101/2023.10.31.564884; this version posted October 31, 2023. The copyright holder for this preprint (which was not certified by peer review) is the author/funder, who has granted bioRxiv a license to display the preprint in perpetuity. It is made available under aCC-BY 4.0 International license.

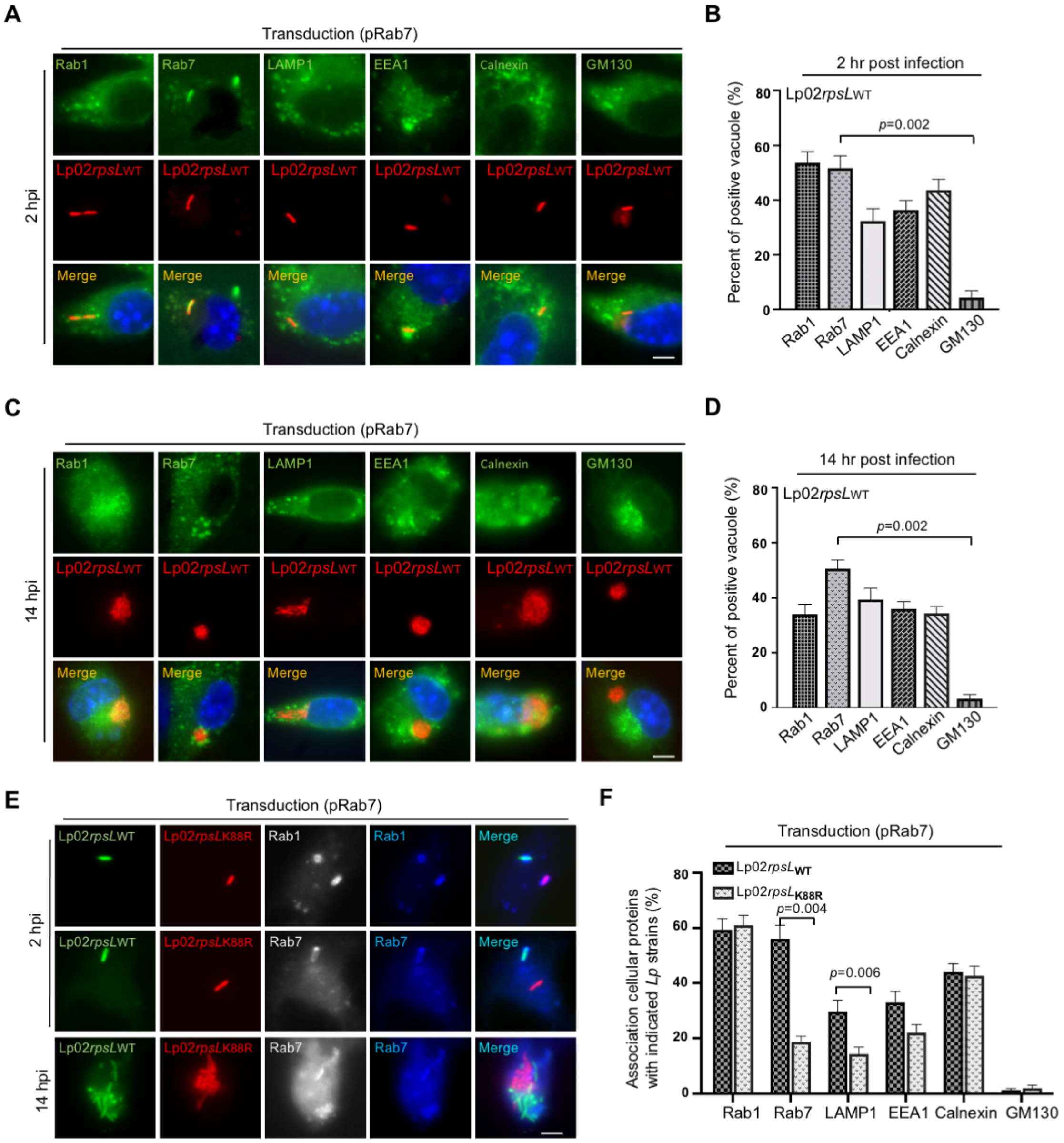

Figure S4 Association of cellular organelles or relevant proteins with LCVs formed by Lp02*rpsL*$_{\mathrm{WT}}$ in BMDMs

(A-D) Representative images of LCVs associated with cellular organelles or relevant proteins in BMDMs infected by strain Lp02*rpsL*$_{\mathrm{WT}}$ for 2 h **(A)** or 14 h **(C).** BMDMs were transduced by lentiviruses that direct Rab7 expression. 2 d post transduction, cells were infected by bacteria at an MOI of 1. Bar, 5 µm. Quantitation of the association of the indicated proteins with LCVs at 2 hpi **(B)** or 14 hpi **(D)**. At least 300 LCVs were counted for each sample.

bioRxiv preprint doi: https://doi.org/10.1101/2023.10.31.564884; this version posted October 31, 2023. The copyright holder for this preprint (which was not certified by peer review) is the author/funder, who has granted bioRxiv a license to display the preprint in perpetuity. It is made available under aCC-BY 4.0 International license.

(E) The distribution of Rab7 in BMDMs co-infected with strains Lp02*rpsL*$_{\mathrm{WT}}$ and Lp02*rpsL*$_{\mathrm{K88R}}$. Note that Rab7 signals on LCVs formed by strain Lp02*rpsL*$_{\mathrm{WT}}$ were stronger than those on strain Lp02*rpsL*$_{\mathrm{K88R}}$. Bar, 5 μm.

(F) Quantitation of the association of relevant proteins with LCVs in BMDMs. Results (mean ± s.e.) were collected from three independent experiments

bioRxiv preprint doi: https://doi.org/10.1101/2023.10.31.564884; this version posted October 31, 2023. The copyright holder for this preprint (which was not certified by peer review) is the author/funder, who has granted bioRxiv a license to display the preprint in perpetuity. It is made available under aCC-BY 4.0 International license.

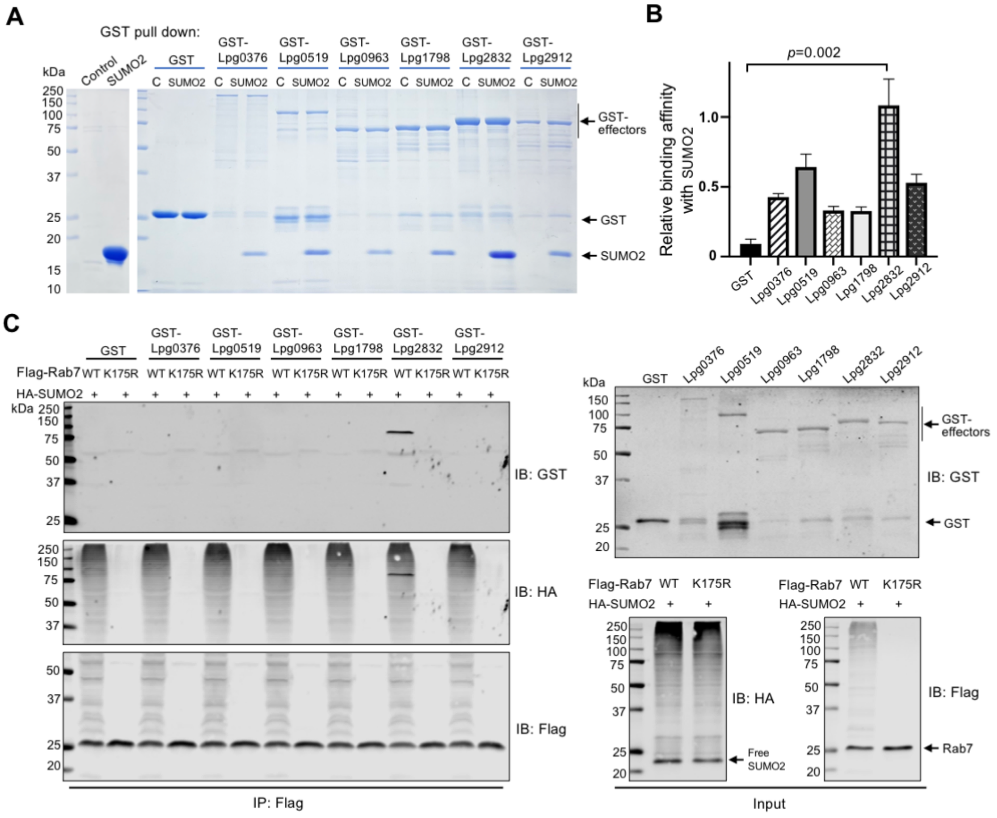

Figure S5 Identification of *L. pneumophila* effectors that contain a predicted SIM involved in binding SUMO and SUMOylated Rab7

(A) Evaluation of the binding by GST pulldown assays. Lysates of *E. coli* expressing the candidate proteins were incubated with GST resins (5%) for 2 h. Washed GST resins coated with candidate effectors were incubated with purified SUMO2 for another 1 h and retained proteins were analyzed by SDS-PAGE and Coomassie brilliant blue staining.

(B) Quantitation of the binding between candidate effectors and SUMO2. The binding affinity was calculated from the band intensity of SUMO2 to GST-effectors. The relative binding affinity was calculated by dividing the binding affinity of each GST-effector by the binding affinity of GST-SulF. Data were collected from three independent experiments and shown is the mean ± s.e.

(C) Immunoprecipitation showing the binding of SUMOylated Rab7 and SulF. HEK293T cells were transfected to express Flag-Rab7 and HA-SUMO2 for 36 h. Beads coated with Flag-SUMO-Rab7 were incubated with recombinant effectors for 1 h and the retained proteins were detected by immunoblotting with appropriate antibodies.

bioRxiv preprint doi: https://doi.org/10.1101/2023.10.31.564884; this version posted October 31, 2023. The copyright holder for this preprint (which was not certified by peer review) is the author/funder, who has granted bioRxiv a license to display the preprint in perpetuity. It is made available under aCC-BY 4.0 International license.

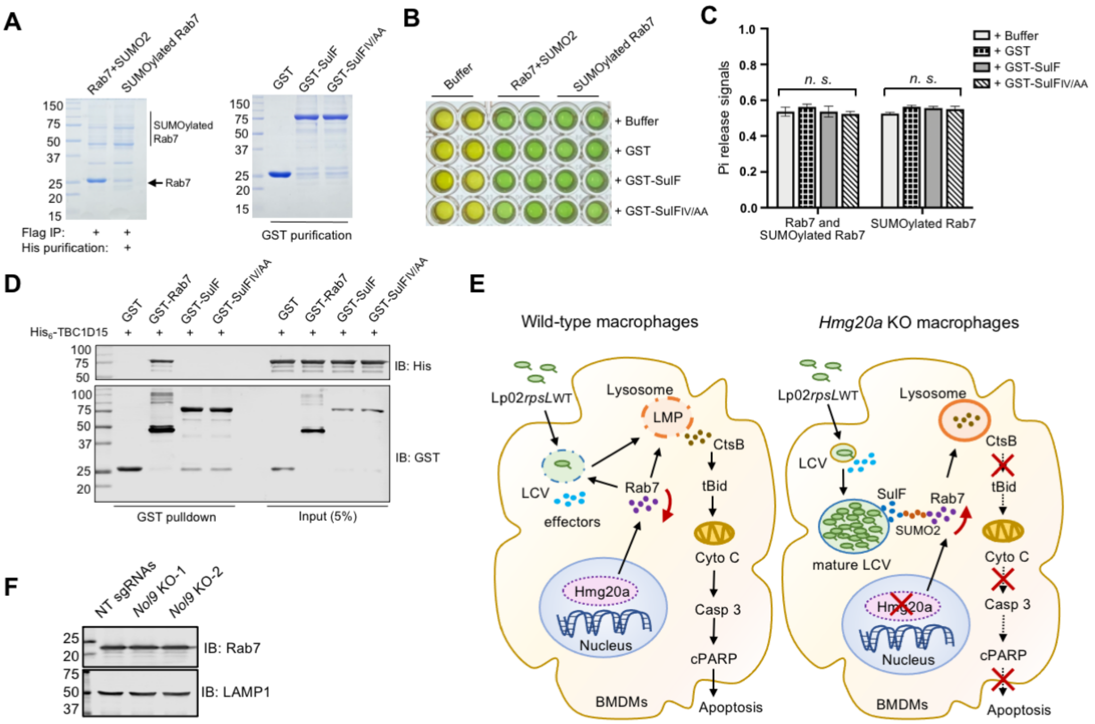

Figure S6 SulF does not impact the GTPase activity of Rab7

(A) SUMO-Rab7 obtained by a two-step purification procedure was evaluated by SDS-PAGE and CBB staining.

(B-C) SulF does not impact the GTPase activity of Rab7. Rab7 or SUMOylated Rab7 were incubated with SulF for 30 min. Then reactions were stopped and released Pi signals at 488nm were recorded using a plate reader. The image of one experiment **(B)** and the activity measured by released free phosphate from three independent experiments mean ± s.e. **(C)** were shown.

(D) SulF did not interact with TBC1D15. GST-Rab7 or GST-SulF (or its SIM-defective mutant) was mixed with His_6-TBC1D15 and the potential protein complexes were captured with GST beads. Retained proteins separated by SDS-PAGE were detected by immunoblotting with the indicated antibodies.

(E) Summary model for SulF-mediated recruitment of SUMOylated Rab7 in Hmg20a knockout macrophages.

(F) *Nol9* knockout did not affect Rab7 expression. Proteins from the indicated cells separated by SDS-PAGE were probed with antibodies specific for Rab7 and LAMP1, respectively.
